# Quasi-Real Time Estimation of Angular Kinematics Using Single-Axis Accelerometers

**DOI:** 10.3390/s130100918

**Published:** 2013-01-15

**Authors:** Alessio Caroselli, Fabio Bagalà, Angelo Cappello

**Affiliations:** 1 Department of Electrical, Electronic and Information Engineering-Guglielmo Marconi, University of Bologna, Viale Risorgimento 2, 40136 Bologna, Italy; E-Mails: alessio.caroselli@gmail.com (A.C.); angelo.cappello@unibo.it (A.C.); 2 Health Sciences and Technologies Interdepartmental Center for Industrial Research (HST-ICIR), University of Bologna, Via Tolara di Sopra 50, 40064 Ozzano Emilia, Bologna, Italy

**Keywords:** inverted pendulum, single-axis accelerometer, second order differential equation, postural sway, unstable non linear system, boundary conditions

## Abstract

In human movement modeling, the problem of multi-link kinematics estimation by means of inertial measurement units has been investigated by several authors through efficient sensor fusion algorithms. In this perspective a single inertial measurement unit per link is required. This set-up is not cost-effective compared with a solution in which a single-axis accelerometer per link is used. In this paper, a novel fast technique is presented for the estimation of the sway angle in a multi-link chain by using a single-axis accelerometer per segment and by setting the boundary conditions through an *ad hoc* algorithm. The technique, based on the windowing of the accelerometer output, was firstly tested on a mechanical arm equipped with a single-axis accelerometer and a reference encoder. The technique is then tested on a subject performing a squat task for the knee flexion-extension angle evaluation by using two single-axis accelerometers placed on the thigh and shank segments, respectively. A stereo-photogrammetric system was used for validation. RMSEs (mean ± std) are 0.40 ± 0.02° (mean peak-to-peak range of 147.2 ± 4.9°) for the mechanical inverted pendulum and 1.01 ± 0.11° (mean peak-to-peak range of 59.29 ± 2.02°) for the knee flexion-extension angle. Results obtained in terms of RMSE were successfully compared with an Extended Kalman Filter applied to an inertial measurement unit. These results suggest the usability of the proposed algorithm in several fields, from automatic control to biomechanics, and open new opportunities to increase the accuracy of the existing tools for orientation evaluation.

## 1. Introduction

The inverted pendulum (IP) is a simple system that finds application in many disciplines of science. Despite its simple nature, it represents a non-linear, unstable and non-minimum phase system, finding several applications in control theory and biomechanics. Achieving stability of an IP has become a common engineering challenge for researchers and the problem has been discussed theoretically by several authors [1–5] and experimentally demonstrated by others [4–7].

There are many examples of the IP model, both man made and found in the natural world. In control theory the challenge of control made the IP system a classic tool in control laboratories [8–12]. The balancing of an IP by moving a cart along a horizontal track is a classic problem in the area of control. Usually one of the state variables of the system which is also the controlled variable, the sway angle, is evaluated from expensive measurement system (e.g., encoder) placed at the pivot joint. The main limitation of this approach is that placing the encoder at the pivot is not always possible.

Arguably the most prevalent example of an IP is a human being. A standing human looks like an IP with the center of mass well above the ground [13–15]. The mechanism to keep balance during the standing posture has intrigued scientists from several fields for a long time [14] and has much significance in clinics. Several authors focused on the estimation of the sway angle since this measurement is related to oscillation of the center of mass of a subject and to the neural control of posture during perturbed and unperturbed stance. However, recent studies [16,17] focused on the contribution of hip and knee to balance control. Given the implications that non-rigidity at the knee and hip may have for the postural control during unperturbed stance, a comprehensive analysis of the ankle, knee and hip movements is required. The analysis revealed that the one-segment IP model is an oversimplification of reality. A multilink model which takes into account thigh, shank and arm-trunk-head segments should be adopted to have a more accurate description of standing balance. From this perspective, the IP model plays the role of the basic element of a multilink chain [18,19]. Kinematics analysis of a multi-link chain should be analyzed using the framework of multiple IPs [16].

In the last years, sensing hardware developments have made available on the market miniaturized inertial (accelerometers and/or gyroscopes) and magnetic sensors. These sensors have found applications in robotics and biomechanics because of their low cost, small size and weight, low power consumption, ease of use and portability. The problem of accurate tracking of orientation by means of these sensors has thus become important in several domains since these wearable sensors can be considered the most valuable opportunity to monitor kinematics and dynamics of human subjects outside specialized laboratories. The problem of solving orientation estimation by using efficient sensor fusion filtering algorithms has been investigated by several authors [20–24] in order to overcome some relevant limitations related to the use of inertial and magnetic sensors one by one:

- orientation estimated by time-integrating, from unknown initial conditions, the signals from a triad of mutually orthogonal uni-axial gyros is prone to errors that grow unbounded over time, due to low-frequency gyro bias drifts;
- it is difficult give a simple interpretation to the accelerometer signals, where the component due to the gravity field (vertical reference) coexists with the component related to the motion of the object;
- nearby ferromagnetic materials are critically disturbing sources when attempts are made to interpret the signals from a tri-axial magnetic sensor as the horizontal reference.

However, the set-up including one inertial measurement unit (IMU) per segment is not cost-effective compared with a solution in which a single-sensor is used. Recently, Bagalà *et al.*[19] showed that it is possible to separate the two different contributions (gravity and inertia) of the accelerometer signal through a bidirectional low-pass filter and a model-based approach. The accuracy in the joint angles estimation using a single-axis accelerometer (SAA) per segment was comparable with that obtained in several previously published studies where two or more sensors per segment were required [25–30].

The aim of this study is to provide a novel technique, faster than that proposed previously [19], based on the Thomas algorithm [31], for the quasi-real time estimation of the sway angle of an IP using (only) one SAA. The algorithm is then extended to a 2-link chain for the estimation of the knee flexion-extension angle of a subject performing a squat task. Furthermore, a comparison with an Extended Kalman Filter (EKF) applied to different sensor configurations was performed.

## 2. Experimental Section

### 2.1. Inverted Pendulum Kinematics

**Figure 1.**
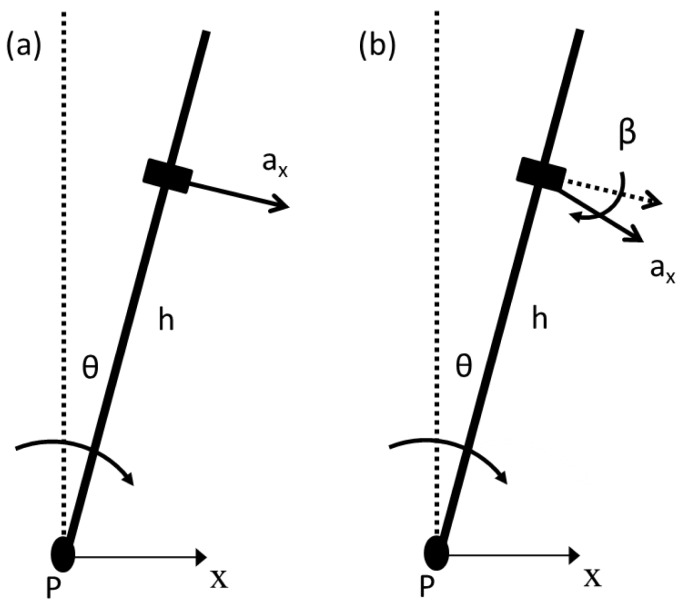
(**a**) IP model and (**b**) IP model with misalignment of the sensitive axis of the SAA.

An IP model in 2D is analyzed. A SAA is placed at height *h* from the pivot point P with the sensitive axis orthogonal to the longitudinal axis of the IP (Figure 1(a)). The accelerometer output, *a_x_*(*t*) can be expressed in the continuous-time domain as the sum of an inertial contribution depending on the angular acceleration component, *α*(*t*) (the second derivative of the sway angle, *θ*(*t*)) and a gravitational term depending on the sway angle, as follows:

(1) $$a_{x}(t)=h\alpha (t)-g\sin\theta (t)$$

where *g* is the gravitational acceleration. Equation (1) is a second order differential equation which has a clear similarity with the equation of motion of the IP: under the assumption of no friction or any other resistance to movement, if *d* is the distance between the center of mass of the pendulum and the pivot, *m* is the mass of the pendulum, *M* is the moment at the pivot and *J* is the moment of inertia, the equation of motion is *M* = *Jα*(*t*) – *mgd*·sin*θ*(*t*). Dividing by *md* the equation of motion $\frac{M}{md}=\frac{J}{md}\cdot \alpha (t)-g\cdot \sin\theta (t)$ becomes similar to Equation (1) since $\frac{M}{md}$ has the dimension of an acceleration and $\frac{J}{md}$ the dimension of a length. Moreover, from a mechanical point of view, the accelerometer is described by a mass-spring system whose behavior is similar to that of a pendulum. The accelerated mass has the same direction of the gravity when the accelerometer is placed on a classical pendulum (stable behavior) and has opposite direction in the case here presented of the IP (unstable behavior).

Several authors [28–30] used an IP and a quasi-static model in which the inertial term in Equation (1) is neglected, so the sway angle can be easily evaluated from the gravitational term and the accelerometer output. This approximation introduces significant errors when the inertial term is not negligible and the frequency bandwidth content of the angular sway is wide.

Unlike the ideal condition of the mathematical model described by Equation (1), the placement of the sensor on a mechanical link potentially introduces some errors due to the non-orthogonality of the sensitive axis of the SAA to the segment. Under the assumption of small misalignment angle, Equation (1) is modified taking into account the projections of the centripetal, tangential and gravity accelerations on the sensitive axis:

(2) $$a_{x}(t)=h\alpha (t)-g\sin\theta (t)+\beta \left[h\omega^{2}(t)-g\cos\theta (t)\right]$$

where *ω*(*t*) represents the angular velocity and *β* describes the SAA non-orthogonality, as shown in Figure 1(b).

In the discrete-time domain, Equation (2) can be rewritten as:

(3) $$\begin{array}{l} a_{x,k}=h\alpha_{k}-g\sin\theta_{k}+\beta \left[h\omega_{k}^{2}-g\cos\theta_{k}\right]\\ k=1,\ldots ,N \end{array}$$

where *N* is the number of samples.

By using the first and second derivative approximations for the angular velocity, *ω*(*t*), and the angular acceleration, *α*(*t*), the relationship between the accelerometer output and the angular sway becomes:

(4) $$\begin{array}{l} a_{x,k}=\frac{h}{T^{2}}(\vartheta_{k+1}-2\theta_{k}+\theta_{k-1})-g\frac{\sin\theta_{k}}{\theta_{k}}\theta_{k}+\beta \left[h{\left(\frac{\theta_{k+1}-\theta_{k-1}}{2T}\right)}^{2}-g\cos\theta_{k}\right]\\ k=2,\ldots ,N-1 \end{array}$$

where *T* is the sample time. Equation (4) can be rewritten as follows:

(5) $$\begin{array}{l} a_{x,k}=\frac{h}{T^{2}}\theta_{k-1}\frac{h}{T^{2}}\theta_{k+1}+\left(-2\frac{h}{T^{2}}-g\frac{\sin\theta_{k}}{\theta_{k}}\right)\vartheta_{k}+\beta \left(\frac{h}{T^{2}}\frac{{\left(\theta_{k+1}-\theta_{k-1}\right)}^{2}}{4}-g\cos\theta_{k}\right)\\ k=2,\ldots ,N-1 \end{array}$$

For a given time window of *W* samples, Equation (5) is a system of *W* − *2* non linear 2nd order differential equations. After setting the two boundary conditions *θ*_1_, *θ_W_*, whose details will be provided in the next section, Equation (5) can be expressed in a matrix form as:

(6) $$\underset{\text{A}}{\underset{︸}{\left[\begin{array}{c} a_{x,2}\\ \vdots \\ a_{x,W-1} \end{array}\right]}}=\underset{\text{C}}{\underset{︸}{\left[\begin{array}{ccccc} C_{2} & B & 0 & \ldots & 0\\ B & C_{3} & \ddots & \ldots & 0\\ 0 & \ddots & \ddots & \ddots & \vdots \\ \vdots & \ddots & \ddots & \ddots & B\\ 0 & \ldots & 0 & B & C_{W-1} \end{array}\right]}}\underset{\theta }{\underset{︸}{\left[\begin{array}{c} \theta_{2}\\ \vdots \\ \theta_{W-1} \end{array}\right]}}+\underset{\text{D}}{\underset{︸}{\left\{B\left[\begin{array}{c} \theta_{1}\\ 0\\ \vdots \\ 0\\ \theta_{W} \end{array}\right]+\frac{\beta B}{4}\left[\begin{array}{c} {\left(\theta_{3}-\theta_{1}\right)}^{2}\\ {\left(\theta_{4}-\theta_{2}\right)}^{2}\\ \vdots \\ {\left(\theta_{W-1}-\theta_{W-3}\right)}^{2}\\ {\left(\theta_{W}-\theta_{W-2}\right)}^{2} \end{array}\right]-g\beta \left[\begin{array}{c} \cos\theta_{2}\\ \vdots \\ \cos\theta_{W-1} \end{array}\right]\right\}}}$$

where $B=\frac{h}{T^{2}}$, $C_{k}=-2B-g\cdot \frac{\sin\theta_{k}}{\theta_{k}}(k=2,\ldots ,W-1)$. Equation (6) can be rewritten in matrix form as **A = Cθ + D** and the sway angle **θ** can be obtained from the accelerometer output vector **A**, through the **C**-matrix inversion as:

(7) $$\theta ={\text{C}}^{-1}(\text{A}-\text{D})$$

The computational cost for the inversion of *N*-square tridiagonal matrix is $\frac{1}{6}N^{3}+O(N^{2})$, which can be reduced to *O*(*N*) by using the tridiagonal matrix algorithm proposed by Thomas [31], which is a simplified form of Gaussian elimination, based on three steps (factorization, forward and backward substitution).

In order to apply the Thomas algorithm for the evaluation of the sway angle, Equation (7) was rewritten in the form of a tridiagonal equation system as:

(8) $$\text{C}\;\theta =\underset{{\text{A}}^{\prime }}{\underset{︸}{\left(\text{A}-\text{D}\right)}}\Rightarrow \text{C}\;\theta ={\text{A}}^{\prime }$$

### 2.2. Algorithm Overview

**Figure 2.**
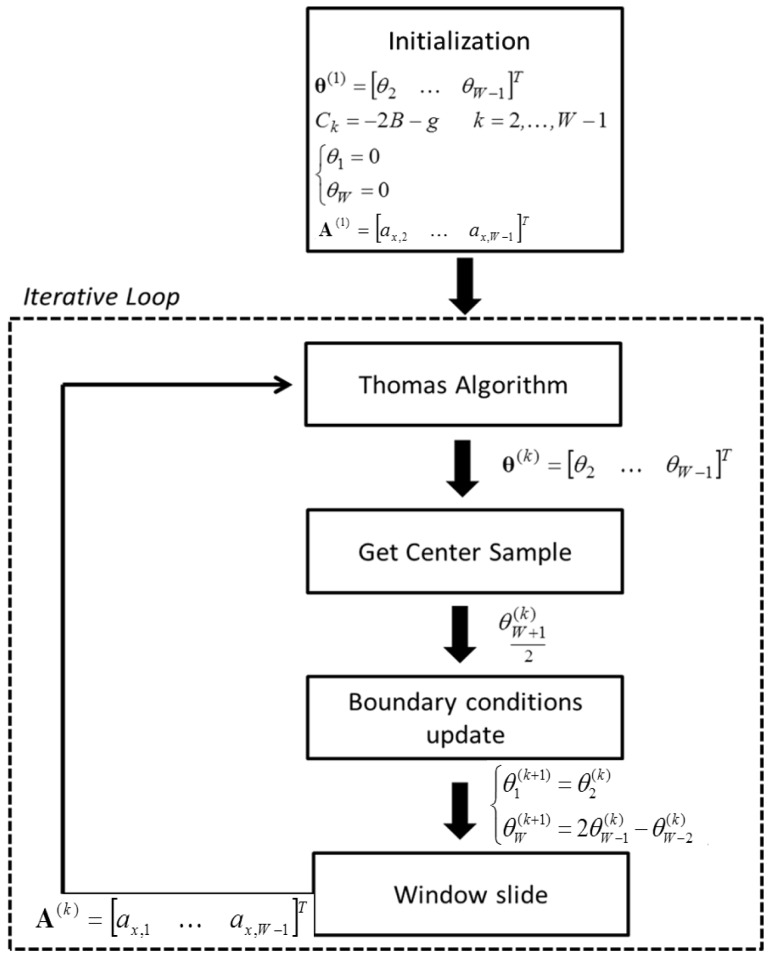
Algorithm structure.

In order to solve Equation (8), a novel algorithm (Figure 2) for the quasi-real time estimation of the sway angle **θ** from the accelerometer output vector **A**, was implemented in Matlab2008. The algorithm is based on the windowing of *W* sample sliding window to the accelerometer output vector **A**. As shown in the right member of Equation (6), the solution of Equation (8) requires setting the two boundary conditions, *θ*_1_, *θ_W_* of the sway angle vector **θ**. They are zeroed when the process starts and are updated, step by step, through the equations described in the following. If the boundary conditions are unknown, as usually occurs in experimental conditions, the angular sway estimate is negatively affected by a transient response at the boundaries. Nevertheless, if the window size is sufficiently large, the central term of the vector **θ**, obtained after solving Equation (8), is largely independent from the boundary conditions.

In the following, the vectors **θ**^(^*^k^*^)^ and **A**^(^*^k^*^)^ represent the sliding window of *W* samples at step *k*. Assuming known the misalignment angle, *β*, the sway angle can be obtained by the following steps (Figure 2):

1. *Initialization*: at the first step, Equation (8) is initialized by zeroing the windowed angle vector **θ**^(1)^ = [*θ*_2_, …, *θ_W_*_−_*_1_*]*^T^* and the two boundary conditions (*θ*_1_ = 0, *θ_W_* = 0). The terms on the diagonal of the matrix **C** are assumed to be constant (*C_k_* = −2*B* – *g*, *k* = 2, …, *W* − 1) by approximating $\frac{\sin\theta_{k}}{\theta_{k}}\approx 1$. The sliding window of the accelerometer output **A**^(1)^ = [*a*_*x*,2_ …, *a*_*x,W*−1_]*^T^* is considered as input.
2. *Thomas algorithm*: the sway-angle vector **θ**^(^*^k^*^)^ is estimated from Equation (8) with *I* iterations by using the Thomas algorithm [31]. A single iteration is enough to have accurate estimates, except for the first cycle, which requires more iterations (*I* = 3) to converge because of the initialization process. Increasing the number of iterations *I* does not significantly improve the estimation accuracy but increases the computational cost.
3. *Get center sample*: as discussed before, at step *k*, the center sample $\theta_{W+1}^{\left(k\right)}/2$ of the estimated vector **θ**^(^*^k^*^)^ is considered as correct estimate, under the assumption of adequately large time windows.
4. *Boundary conditions update*: according to the result provided by the Thomas algorithm at the previous step, at step *k* > 1 the two boundary conditions are set as the first element of the vector **θ**^(^*^k^*^)^ (left boundary) and as the linear interpolation of the last two elements of the vector **θ**^(^*^k^*^)^ (right boundary) as follows:
  $$\{\begin{array}{ll} \begin{array}{l} \theta_{1}^{\left(k+1\right)}=\theta_{2}^{\left(k\right)}\\ \theta_{W}^{\left(k+1\right)}=2\theta_{W-1}^{\left(k\right)}-\theta_{W-2}^{\left(k\right)} \end{array} & k=2,\ldots ,N-1 \end{array}$$
  The terms of the matrix **C** are updated with the angle vector elements.
5. *Window Slide*: the input vector **A**^(^*^k^*^)^ is updated by adding the new sample at the end of the vector and deleting the first one.

Steps (2–5) are iterated for the total length of the accelerometer output.

The algorithm's computational cost, *C_T_* can be expressed as:

(9) $$C_{T}=f_{s}(N-W)\bar{I}C_{i}$$

where *C_i_* is the computational cost to process *W* samples by the Thomas algorithm, *Ī* the average number of iterations per window and *N* the total number of samples of the signal to be processed.

The computational cost is therefore reduced with respect to the offline method previously published [19], based on the bidirectional filtering of the accelerometer output. In a linear case (small sway angles), the similarity between the quasi-real time algorithm and the previously published method is demonstrated in Appendix A.

### 2.3. Mechanical Inverted Pendulum

**Figure 3.**
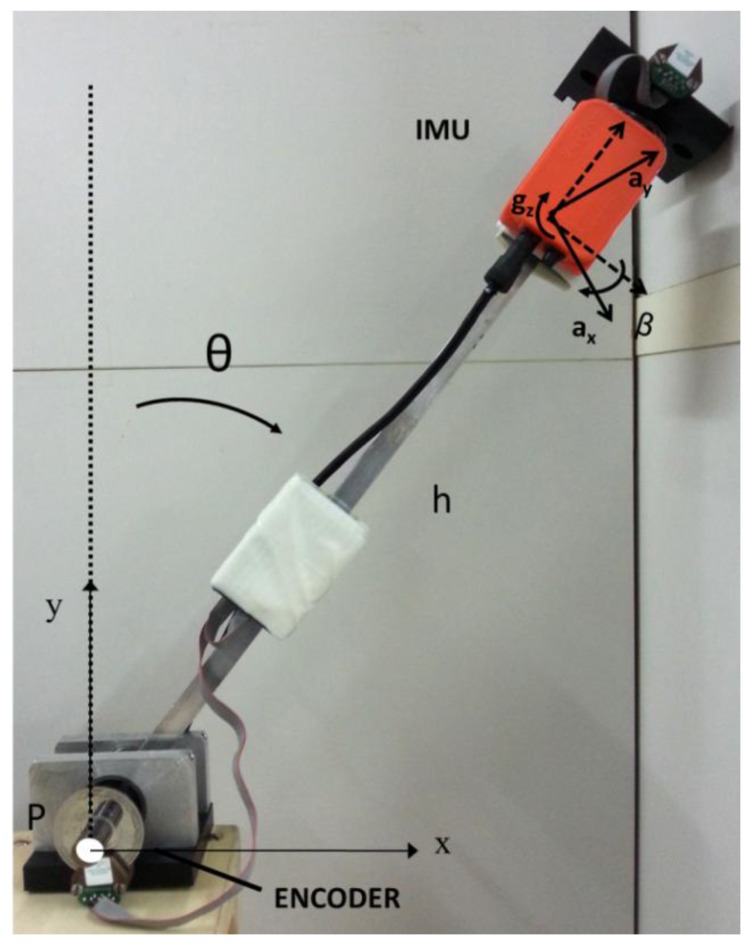
Mechanical Inverted Pendulum.

To test the method, the same experimental setup used in [19] was adopted. An aluminum rectangular link was used as an IP driven by hand to sway with a fixed pivot point. The frequency content of the angular sway is [0–2] Hz. Five trials were performed. The mechanical arm was equipped with an absolute encoder (Gurley, mod. 7700, resolution 19 bit, Gurley Precision Instruments, Troy, NY, USA) and an IMU (MTx, Xsens Technologies, Eschede, The Netherlands), consisting of a tri-axial accelerometer, a tri-axial gyroscope and a tri-axial magnetometer, placed at height *h* = 0.20 m from the pivot point P (Figure 3).

The IMU's outputs were acquired at *f_s_* = 50 Hz sampling frequency. To test the algorithm presented in this paper, only the accelerometer output related to the axis orthogonal to the mechanical arm, *a_x_* was considered as input. A preliminary calibration, in which the encoder output was used as reference, was performed to estimate the two geometric parameters *h* and *β*. The two parameters were estimated by minimizing the RMSE between the encoder output and the estimated angle.

In order to test the robustness of the method proposed in this paper, results in terms of RMSE were compared with:

- those obtained by using the offline method proposed in [19], where a bidirectional low-pass filtering of the accelerometer output, with a cut-off frequency depending on the position of the sensor, was implemented;
- those obtained by using an EKF applied to the outputs of the IMU (Figure 3), in particular to the accelerometers with sensitive axis orthogonal and along the mechanical arm, *a_x_* and *a_y_*, and to the output of the gyroscope with sensitive axis orthogonal to the plane of the movement, *g_z_* as discussed in the next section.

### 2.4. Extended Kalman Filter

The outputs of the accelerometers along the *x* and *y* directions and the output of the gyroscope with the sensitive axis orthogonal to the plane of the movement (*z*) are expressed as follows:

(10) $$\begin{array}{l} a_{x,k}=h\alpha_{k}-g\sin\theta_{k}+\beta \left[h\omega_{k}^{2}-g\cos\theta_{k}\right]\\ a_{y,k}=-h\omega_{k}^{2}+g\cos\theta_{k}+\beta \left[h\alpha_{k}-g\sin\theta_{k}\right]\\ g_{z,k}=\omega_{k}\\ k=1,\ldots ,N \end{array}$$

The main equations of the EKF are shown in Equation (11), where ***w****_k_* and ***v****_k_* represent the process and measurement noise, assumed to be independent, white and with normal probability distribution with a process noise covariance **Q** and a measurement noise covariance **R**. More details about the algorithm are presented elsewhere [32,33]. The state-space model of the EKF is described by the process and measurement model equations:

(11) $$\begin{array}{l} {\text{x}}_{k+1}=\text{A}{\text{x}}_{k}+{\text{w}}_{k}\\ {\text{z}}_{k}=h({\text{x}}_{k})+{\text{v}}_{k} \end{array}$$

The state vector of the EKF, **x**(*k*)_[3×1]_, at every sampled instant of time *k*, was defined as $\text{x}(k)={\left[\theta (k)\;\omega (k)\;\alpha (k)\right]}^{T}$.

In the discrete-time domain the predicted state at the instant *k* + *1* was obtained as:

(12) $${\text{x}}_{k+1}=\underset{{\text{A}}_{\left[3\times 3\right]}}{\underset{︸}{\left[\begin{array}{ccc} 1 & \text{T} & \overset{T^{2}}{\;}/\underset{2}{\;}\\ 0 & 1 & \text{T}\\ 0 & 0 & 1 \end{array}\right]}}{\text{x}}_{k}$$

The vector of the measurements **z**(*k*)_[3×1]_ was defined considering the outputs of the sensors as $\text{z}(k)={\left[a_{x}(k)\;a_{y}(k)\;g_{z}(k)\right]}^{T}$.

According to (10), the output vector is related to the state vector through the non-linear relationship **z**(*k*) = **h**(**x***_k_*). The output matrix **H**(*k*)_[3×3]_ which relates the measurements **z**(*k*)_[3×1]_ to the state **x**(*k*) was thus obtained evaluating the Jacobian matrix of *h*(**x***_k_*) with respect to the state vector.

The process covariance matrix **Q**_[3×3]_ was defined under the assumption that process noise affects the jerk only and there are no correlations between the jerk noise sequences. **Q**_[3×3]_ has therefore only one non-zero element *Q*(3,3) set to 10^−3^. The measurement noise covariance matrix **R**_[3×3]_ was defined considering the noise which affects the gyroscope and accelerometer outputs. Since correlations between noise of the sensors were assumed to be zero, the covariance matrix was put in the form 10^−8^·I_[3×3]_. All these tuning parameters were defined after an optimization procedure in which the encoder was assumed as validation standard. In order to start the filtering procedure, initial estimate of the state vector was zeroed whereas the initial estimate of the error covariance matrix **P**_[3×3]_ was set equal to the identity matrix.

In order to compare the performance of the window-based algorithm proposed in this study with the EKF, seven different configurations of the EKF were implemented by considering the following measurement vectors:

- $\text{z}(k)={\left[a_{x}(k)\;a_{y}(k)\;g_{z}(k)\right]}^{T}$;
- $\text{z}(k)={\left[a_{x}(k)\;g_{z}(k)\right]}^{T}$;
- $\text{z}(k)={\left[a_{y}(k)\;g_{z}(k)\right]}^{T}$;
- $\text{z}(k)={\left[a_{x}(k)\;a_{y}(k)\right]}^{T}$;
- *z*(*k*) = *a_x_*(*k*);
- *z*(*k*) = *a_y_*(*k*);
- *z*(*k*) = *g_z_*(*k*);

For each case, the output matrix **H**(*k*) and the measurement noise covariance matrix **R** were adjusted to the dimension of the measurement vector. The tuning parameters, **Q** and **R**, were optimized for each case to have the best performance in terms of RMSE of the angle estimation.

### 2.5. 2-Link Chain: Knee Flexion-Extension Angle Evaluation

In order to show an application of the algorithm proposed in this paper and its extension to a multi-link model for human movement kinematics estimation, the knee flexion-extension angle of a subject performing a squat task was evaluated.

**Figure 4.**
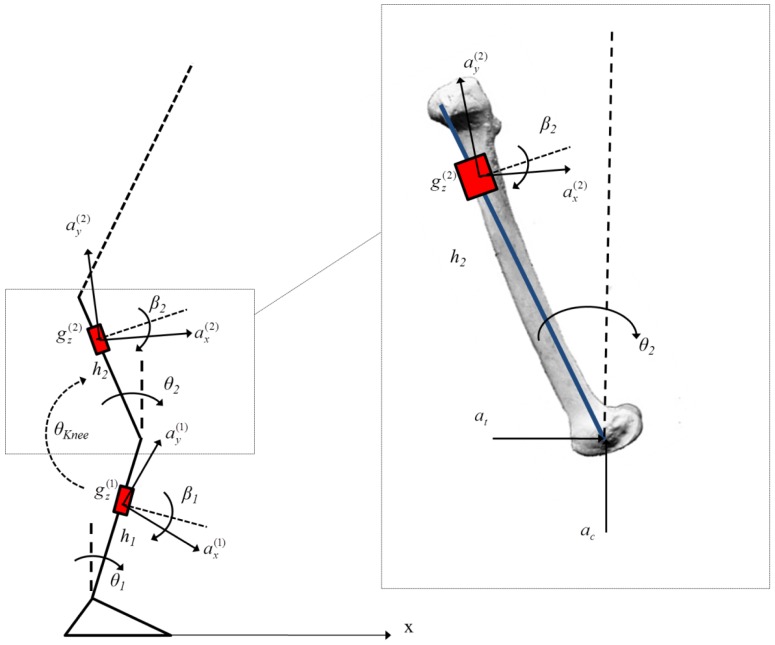
Two-links model for the knee flexion-extension angle estimation.

The method is tested on one subject (male, 29 years old, weight 74 kg, height 176 cm) who participated after giving his informed consent. In order to estimate the thigh and shank sway in the sagittal plane during squat tasks [34], a 2-link biomechanical model is introduced for the two segments (Figure 4). The feet are supposed rigidly connected to the ground; the ankle and the knee joints are represented as two hinge joints and the shank (segment 1, length *l_1_* = 0.41 m and the thigh (segment 2) are modeled as two rigid segments. The HAT kinematics has not been investigated in the present study. The subject was asked to perform a repetition of squat exercises for one minute with arms folded, keeping his movement in the sagittal plane. Five trials were performed. In order to estimate the knee flexion-extension angle, two IMU's (MTx, Xsens Technologies) were placed at measured heights *h_1_* = 0.27 m, *h_1_* = 0.19 m with respect to the ankle and knee joint, respectively. Each of the two sensors was placed on a rhomboid rigid plate and mounted on the skin at the lateral side of the thigh and the shank by using three hook-and-loop fastener belts.

Four reflective markers were placed on the vertices of each plate, and a stereo-photogrammetric system (SMART eMOTION, BTS) was used for calibration of the geometric parameters (*h*_1_, *h*_2_, *l*_1_, *β*_1_, *β*_2_). The two reference angles with respect to the vertical were evaluated through the 2D Singular Value Decomposition (SVD) method [35,36]. Stereo-photogrammetric and inertial data were sampled at 100 Hz.

The outputs of the sensor placed on the thigh can be evaluated by modifying Equation (10) by adding the projection of the accelerations *a_t_* and *a_c_* on the measurement axis of the sensor.

These two contributions can be evaluated considering the second derivative of the position of the knee joint with respect to the ankle joint:

(13) $$\{\begin{array}{l} {a_{t,x}=l_{1}\frac{d^{2}[\sin\theta_{1}(t)]}{dt^{2}}|}_{t=kT}\approx l_{1}\frac{\sin\theta_{1,k+1}-2\sin\theta_{1,k}+\sin\theta_{1,k-1}}{T^{2}}\\ {a_{c,x}=l_{1}\frac{d^{2}[\cos\theta_{1}(t)]}{dt^{2}}|}_{t=kT}\approx l_{1}\frac{\cos\theta_{1,k+1}-2\cos\theta_{1,k}+\cos\theta_{1,k-1}}{T^{2}} \end{array}$$

The accelerometer and gyroscope outputs placed on the thigh can thus be expressed as follows:

(14) $$\begin{array}{l} a_{x,k}^{\left(2\right)}=h_{2}\alpha_{2.k}-g\sin\theta_{2.k}+\beta_{2}\left[h_{2}\omega_{2,k}^{2}-g\cos\theta_{2.k}\right]+a_{t,k}\cos\left(\theta_{2,k}+\beta_{2}\right)-a_{c,k}\sin\left(\theta_{2,k}+\beta_{2}\right)\\ a_{y,k}^{\left(2\right)}=-h_{2}\omega_{2,k}^{2}+g\cos\theta_{2.k}+\beta_{2}\left[h_{2}\alpha_{2.k}-g\sin\theta_{2.k}\right]+a_{t,k}\sin\left(\theta_{2,k}+\beta_{2}\right)+a_{c,k}\cos\left(\theta_{2,k}+\beta_{2}\right)\\ g_{z,k}^{\left(2\right)}=\omega_{2,k}\\ k=1,\ldots ,N \end{array}$$

where the Equation (A2) indicates that Equation (14) are referred to the thigh segment.

The accelerometer and gyroscope outputs placed on the shank segment are expressed as Equation (10) as follows:

(15) $$\begin{array}{l} a_{x,k}^{\left(1\right)}=h_{1}\alpha_{1,k}-g\sin\theta_{1,k}+\beta_{1}\left[h_{1}\omega_{1,k}^{2}-g\cos\theta_{1,k}\right]\\ a_{y,k}^{\left(1\right)}=-h_{1}\omega_{1,k}^{2}+g\cos\theta_{k}+\beta_{1}\left[h_{1}\alpha_{1,k}-g\sin\theta_{1,k}\right]\\ g_{z,k}^{\left(1\right)}=\omega_{1,k}\\ k=1,\ldots ,N \end{array}$$

The accelerometers outputs $a_{x,k}^{\left(1\right)}$, $a_{x,k}^{\left(2\right)}$ are used as inputs to the Thomas algorithm. In particular, for the second link, the sway angle can be obtained from (7) as **θ** = **C**^−1^(**A**−**D**′) where **D**′ is:

(16) $${\text{D}}^{\prime }=\text{D}+\left[\begin{array}{c} a_{t,k}\cos(\theta_{2,2}+\beta_{2})-a_{c,2}\sin(\theta_{2,2}+\beta_{2})\\ \vdots \\ a_{t,W-1}\cos(\theta_{2,W-1}+\beta_{2})-a_{c,W-1}\sin(\theta_{2,W-1}+\beta_{2}) \end{array}\right]$$

In addition, the IMU's outputs Equations (14) and (15) were considered as the measurement vector for the EKF and, as described in the previous section, seven different configurations were considered:

- $\text{z}(k)={\left[a_{x}^{\left(1\right)}(k)\;a_{y}^{\left(1\right)}(k)\;g_{z}^{\left(1\right)}(k)\;a_{x}^{\left(2\right)}(k)\;a_{y}^{\left(2\right)}(k)\;g_{z}^{\left(2\right)}(k)\right]}^{T}$;
- $\text{z}(k)={\left[a_{x}^{\left(1\right)}\;g_{z}^{\left(1\right)}\;a_{x}^{\left(2\right)}\;g_{z}^{\left(2\right)}\right]}^{T}$;
- $\text{z}(k)={\left[a_{y}^{\left(1\right)}\;g_{z}^{\left(1\right)}\;a_{y}^{\left(2\right)}\;g_{z}^{\left(2\right)}\right]}^{T}$;
- $\text{z}(k)={\left[a_{x}^{\left(1\right)}\;a_{y}^{\left(1\right)}\;a_{x}^{\left(2\right)}\;a_{y}^{\left(2\right)}\right]}^{T}$;
- $\text{z}(k)={\left[a_{x}^{\left(1\right)}(k)\;a_{x}^{\left(2\right)}(k)\right]}^{T}$;
- $\text{z}(k)={\left[a_{y}^{\left(1\right)}(k)\;a_{y}^{\left(2\right)}(k)\right]}^{T}$;
- $\text{z}(k)={\left[g_{z}^{\left(1\right)}(k)\;g_{z}^{\left(2\right)}(k)\right]}^{T}$;

The knee flexion-extension angle was evaluated from the shank and thigh angles, **θ***_shank_* and **θ***_thigh_* respectively, as:

(17) $$\theta_{\text{knee}}=\pi -(\theta_{\text{shank}}-\theta_{\text{thigh}})$$

RMSEs between the flex-extension angles estimated by (1) the algorithm proposed in this paper, (2) the EKF in the seven different configurations described above, were evaluated with reference to the stereo-photogrammetric measurements.

## 3. Results

### 3.1. Mechanical Inverted Pendulum

Five trials were performed. For each of them, the calibration parameters were estimated (mean ± std): the distance *h* = 0.20 ± 0.00 m of the origin of the sensor reference system to the pivot point (the measured distance equals 0.20 m), and the angle β = −1.24 ± 0.07°, related to the non-orthogonality of the measurement axis of the SAA to the link. These values were then used along the sensor data to predict the sway angle which was compared with the encoder angle. RMSEs, averaged over the five trials, were computed and compared with those obtained in [19] and those obtained by using the EKF in seven different cases. Peak-to-peak range (mean ± std), averaged over the five trials, is 147.2 ± 4.9°. Results are reported in Table 1.

**Figure 5.**
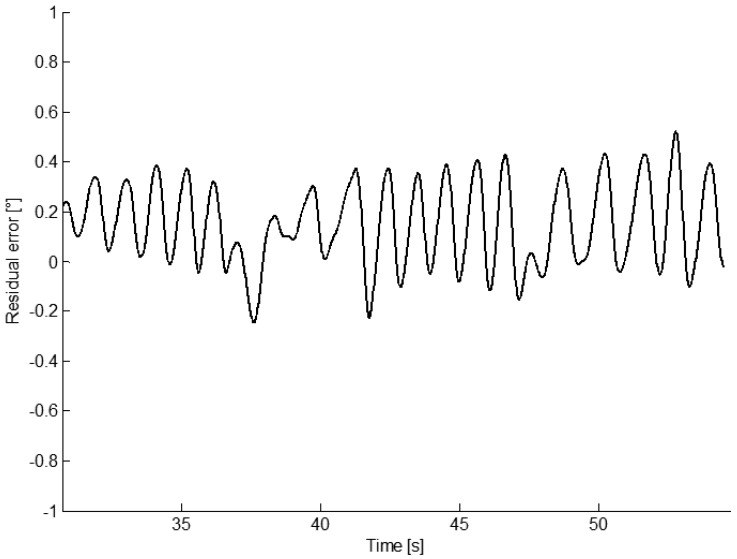
Residual error between the encoder output and sway angle estimated from the accelerometer output in the mechanical inverted pendulum.

In Figure 5 the residual error between the estimated sway angle with the algorithm proposed in this paper and the encoder angle. The RMSE obtained by the quasi-real time algorithm is slightly greater than the RMSE obtained with the offline algorithm proposed by Bagalà *et al.*[19], but with lower standard deviation. Moreover, the proposed algorithm improves the previous method [19] by significantly reducing the computational cost (from 10–15 iterations to a single iteration). RMSEs obtained with the EKF are greater than the RMSE obtained with the algorithm proposed in this paper in all the seven configurations analyzed. In particular, RMSEs are comparable if at least two sensors are used. The best performance is obtained by fusing the outputs of the two accelerometers and the gyroscope but the RMSE dramatically increases when a single sensor is used, since integration errors are not compensated by fusing measurements from different sensors.

The time required by the algorithm was about 170 ms to process 50 s of acquisition with a window of 100 samples, by using a DELL Studio 1,535 computer (processor Intel Core 2 Duo T9300, 2.50 GHz, 4 GB Memory, OS: Windows Vista 32 bit).

**Figure 6.**
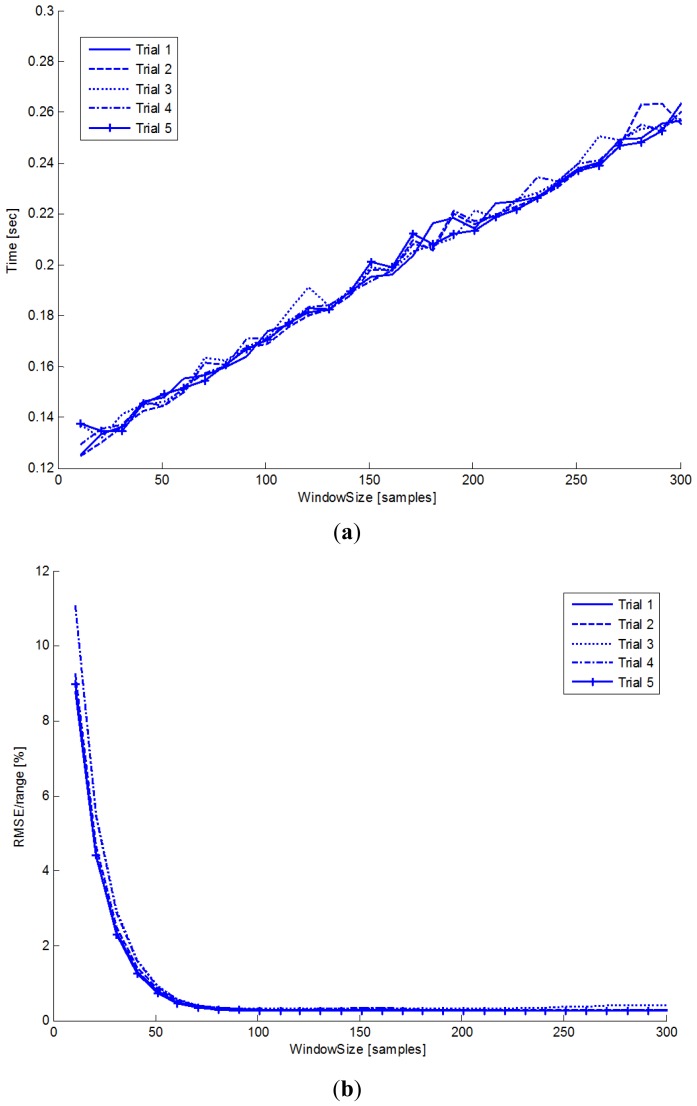
(**a**) Window size *vs.* algorithm speed. (**b**) Window size *vs.* RMSE [%].

A good trade-off between the algorithm's speed and accuracy may be obtained by using Figure 6 which shows the effect of the window size *W* on the time required to process 50 s of the signal in Figure 6(a), and on the percentage ratio between RMSE and peak-to-peak range in Figure 6(b).

It is trivial to note that high values of *W* increase the computational cost because the size of the matrix **C** to be solved by the Thomas algorithm increases.

The RMSE shows an exponential dependence on the window size with a significant reduction for a window duration defined by the time constant of the system in Equation (1). For a bidirectional 1st order filter, the transient effect is negligible if the window size is at least 9.2*τ* where $\tau =\sqrt{h/g^{\prime }}$ and *g*′ = *g*·*sinθ/θ*. Assuming *h* = 0.20 m, a maximum value of the sway equal to π/3 and a mean value of the term *sinθ/θ* ≈ 0.8 the window size should be *W* = 9.2*τfs* ≈ 73 samples. This is confirmed by Figure 6(b) where the RMSE significantly decreases when the window size exceeds 100 samples. Furthermore, as shown in Figure 6(a), the algorithm's computational cost linearly increases with the window size as one would expect: according to (9), if *N* ≫ *W*, *C_T_* ∞ *C_i_* and the computational cost *C_i_* is *O*(*W*) for tridiagonal matrix inversion using the Thomas algorithm. Increasing the window size over 100 samples does not provide significant variation in terms of RMSE but produces an increase of the computational cost.

### 3.2. Knee Flex-Extension Angle

The mean values and the standard deviations of the calibration parameters for the five trials, obtained by minimizing sway angle errors between stereo-photogrammetric and inertial sensor data, are (mean ± std): *h*_1_ = 0.20 ± 0.01 m, *h*_2_ = 0.22 ± 0.00 m, *l*_1_ = 0.40 ± 0.06 m, and *β*_1_ = −8.98 ± 0.24°, *β*_2_ = −2.25 ± 0.71°. These parameters are then used to predict angular sway by using the two accelerometer outputs, $a_{x,k}^{\left(1\right)}$, $a_{x,k}^{\left(2\right)}$, for the quasi-real time algorithm, and by using the IMUs outputs for the EKF, as described in the Section 2.4. RMSEs (mean ± std) are reported in Table 2. Peak-to-peak range (mean ± std), averaged over the five trials, is 59.29 ± 2.02°.

**Figure 7.**
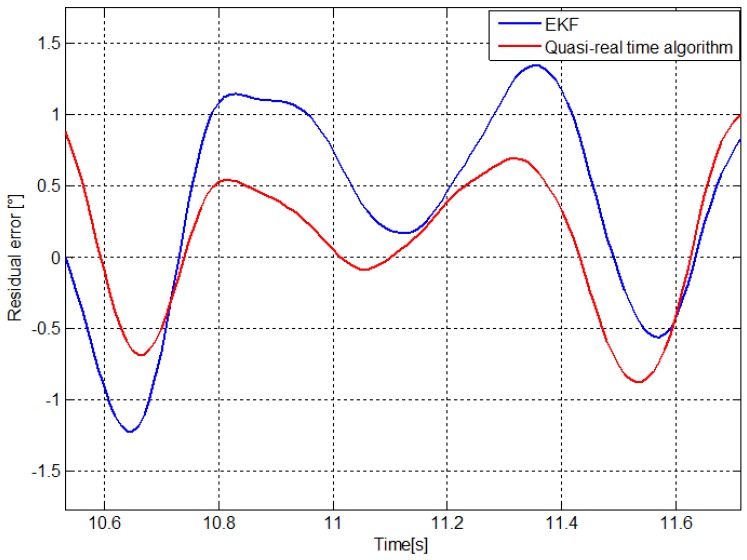
Residual error of the knee flexion-extension angle estimated by the quasi-real time algorithm and the EKF fusing accelerometers and gyroscope outputs. The angle estimated by stereo-photogrammetry was used as reference.

RMSEs reported in Table 2 confirm the results obtained in the mechanical inverted pendulum. The quasi-real time algorithm provides an RMSE comparable with previous published method [19] and lower than that obtained using an EKF in the best configuration where accelerometers and gyroscope outputs are fused. Figure 7 shows residual error of the knee flexion-extension angle during a squat cycle evaluated using the quasi-real time algorithm and the EKF in the best case. Stereo-photogrammetric angle was used as reference.

## 4. Discussion and Conclusions

This paper suggests a novel method for the quasi-real time estimation of the sway angle in an IP model by using one SAA per segment. The relationship between the sway angle and the SAA output is described by a second order differential equation which is solved by applying the Thomas algorithm [31] to the sliding window of the accelerometer output and by setting the boundary conditions of the sway angle through an *ad hoc* algorithm. The method allows the 2D orientation estimation of the IP overcoming the limitations of the double-integration of the accelerometer output, recently discussed in [20]. The method is successfully tested on a mechanical IP providing accurate estimation of the sway angle compared with the encoder output. In addition, the method is extended to a 2-link chain for an application in human movement analysis. The knee flexion-extension angle of a subject performing a squat task was evaluated by using the outputs of two SAAs, placed on the thigh and shank segments, and successfully compared with a stereo-photogrammetric system. The extension to a multi-link chain is straightforward. Our method is further compared with an EKF applied to the IMU's outputs both on the mechanical IP and the human subject. The proposed technique, based on the Thomas algorithm, is more accurate than the EKF as confirmed by the results reported in Tables 1 and 2. According with these considerations the quasi-real time algorithm applied to the output of a SAA represents a cost-effective solution when multi-link kinematics estimation is required: using one IMU per segment is much expensive than using a SAA per segment.

It is worth noting that the geometric parameters (position of the sensor with respect the pivot point, misalignment angle, segment lengths) are estimated by using encoder outputs or stereo-photogrammetric system as validation instruments only in a preliminary calibration phase. After this calibration the parameters are used to predict the sway angle with the algorithm proposed in the paper using inertial sensor only. As shown in the Results section, the estimated segment lengths and the position of the sensor with respect to the pivot are quite similar to those measured by hand. The misalignment angles are lower than 9° and neglecting the misalignment does not significantly increase the RMSE.

The method presented in this paper improves the previous method [19] by significantly reducing the computational cost (from 10–15 iterations to a single iteration). Furthermore, the algorithm is quasi-real time since it provides the estimated angles with a delay of *W/2f_s_* s, while the previous proposed algorithm [19] is offline since it requires the whole accelerometer signal to be processed.

The performance of the estimation method depends on the window size. Figure 6(a,b) provides useful information about the window size, the algorithm speed and the RMSE according to the specifications required. If the speed of the algorithm and the real time performance are the most important requirements, choosing a short time window reduces the computational cost and the angular sway latency. Nevertheless, a short time window reduces the accuracy of the estimation because of the time required for the exhausting the transient effect. For these reasons, if the accuracy is the main specification, increasing the window size provides the best results in terms of RMSE. The sway angle is always estimated with a delay equal to half of the time window.

The results suggest possible applications in various fields, from automatic control to bioengineering. Mainly in biomechanics, the algorithm proposed in this paper may speed up the procedure for the kinematics evaluation of human movement with a cost-effective set-up. An IMU integrating accelerometers and gyroscopes is more expensive and requires the use of a more complex HW/SW architecture for efficient signal processing. These costs increase even more with a multilink chain where one sensor per segment is required. One of the limitations of this study is the quasi-real time implementation due to the intrinsic delay imposed by the window size. Future studies will be addressed to this issue.

## Figures and Tables

**Table 1.** Averaged RMSEs for the mechanical IP.

**Tested algorithms**	**RMSE [°]** **mean ± std**
**Quasi-real time algorithm**	0.40 ± 0.02
**Bagalà*et al.* algorithm** [19]	0.39 ± 0.05
**EKF:** $\text{z}(k)={\left[a_{x}(k)\;a_{y}(k)\;g_{z}(k)\right]}^{T}$	0.45 ± 0.05
**EKF:** $\text{z}(k)={\left[a_{x}(k)\;g_{z}(k)\right]}^{T}$	0.46 ± 0.05
**EKF:** $\text{z}(k)={\left[a_{y}(k)\;g_{z}(k)\right]}^{T}$	2.12 ± 0.15
**EKF:** $\text{z}(k)={\left[a_{x}(k)\;a_{y}(k)\right]}^{T}$	20.64 ± 12.48
**EKF:** *z*(*k*) = *a_x_*(*k*)	26.71 ± 0.70
**EKF:** *z*(*k*) = *a_y_*(*k*)	41.83 ± 7.50
**EKF:** *z*(*k*) = *g_z_*(*k*)	8.87 ± 6.92

**Table 2.** Averaged RMSEs for the knee flex-extension angle.

**Tested algorithms**	**RMSE [°]** **mean ± std**
**Quasi-real time algorithm**	1.01 ± 0.11
**Bagalà *et al.* algorithm** [19]	0.95 ± 0.48
**EKF:** $\text{z}(k)={\left[a_{x}(k)\;a_{y}(k)\;g_{z}(k)\right]}^{T}$	2.43 ± 0.76
**EKF:** $\text{z}(k)={\left[a_{x}(k)\;g_{z}(k)\right]}^{T}$	2.46 ± 0.62
**EKF:** $\text{z}(k)={[a_{y}(k)\;g_{z}(k)]}^{T}$	2.83 ± 0.24
**EKF:** $\text{z}(k)={\left[a_{x}(k)\;a_{y}(k)\right]}^{T}$	37.13 ± 18.28
**EKF:** *z*(*k*) = *a_x_*(*k*)	19.31 ± 0.64
**EKF:** *z*(*k*) = *a_y_*(*k*)	43.27 ± 5.05
**EKF:** *z*(*k*) = *g_z_*(*k*)	3.95 ± 2.59

## Appendix A

For the sake of clarity, Equation (A1) is linearizable under the assumption of small angles and the sway angle *θ*(*t*) can be rewritten in the Laplace domain as sum of two first-order low-pass filters (*F_F_*, forward filter; *B_F_*, backward filter) applied to the accelerometer output:

(A1) $$\begin{array}{cc} \theta (s)=-\frac{a(s)}{2g}\left(\underset{F_{F}}{\underset{︸}{\frac{1}{1+\tau s}}}+\underset{B_{F}}{\underset{︸}{\frac{1}{1-\tau s}}}\right) & \tau =\sqrt{\frac{h}{g}} \end{array}$$

In the discrete-time domain, assuming *θ⃗k* the signal obtained after the forward filtering and *θ⃖k* the signal after the backward filtering of the accelerometer output, Equation (A1) can be split in the two following equations:

(A2) $$\{\begin{array}{c} {\vec{\theta }}_{k}=-\frac{a_{k}}{2g}+\tau \frac{{\vec{\theta }}_{k+\frac{1}{2}}-{\vec{\theta }}_{k-\frac{1}{2}}}{T}\\ {\overset{\leftarrow }{\theta }}_{k}=-\frac{a_{k}}{2g}-\tau \frac{{\overset{\leftarrow }{\theta }}_{k+\frac{1}{2}}-{\overset{\leftarrow }{\theta }}_{k-\frac{1}{2}}}{T} \end{array}$$

By summing up and subtracting the two filtered signals of Equation (A2) we got:

(A3) $$\{\begin{array}{l} {\vec{\theta }}_{k}+{\overset{\leftarrow }{\theta }}_{k}=-\frac{a_{k}}{g}+\frac{\tau }{T}\left({\vec{\theta }}_{k+\frac{1}{2}}-{\vec{\theta }}_{k-\frac{1}{2}}-{\overset{\leftarrow }{\theta }}_{k+\frac{1}{2}}+{\overset{\leftarrow }{\theta }}_{k-\frac{1}{2}}\right)=\theta_{k}\\ {\vec{\theta }}_{k}-{\overset{\leftarrow }{\theta }}_{k}=-\frac{\tau }{T}\left({\vec{\theta }}_{k+\frac{1}{2}}-{\vec{\theta }}_{k-\frac{1}{2}}+{\overset{\leftarrow }{\theta }}_{k+\frac{1}{2}}-{\overset{\leftarrow }{\theta }}_{k-\frac{1}{2}}\right)=-\frac{\tau }{T}\left(\theta_{k+\frac{1}{2}}-\theta_{k-\frac{1}{2}}\right) \end{array}$$

By manipulating Equation (A3), the forward and backward filtered angles can be expressed as function of the sway angle as follows:

(A4) $$\{\begin{array}{l} {\vec{\theta }}_{k}=\frac{1}{2}\left[\theta_{k}+\frac{\tau }{T}\left(\theta_{k-\frac{1}{2}}-\theta_{k+\frac{1}{2}}\right)\right]\\ {\overset{\leftarrow }{\theta }}_{k}=\frac{1}{2}\left[\theta_{k}-\frac{\tau }{T}\left(\theta_{k-\frac{1}{2}}-\theta_{k+\frac{1}{2}}\right)\right] \end{array}$$

By substituting the two terms of Equation (A4) in the first of Equation (A3), it is trivial to obtain:

(A5) $$\frac{h}{T^{2}}\theta_{k-1}-2(g+\frac{h}{T^{2}})\theta_{k}+\frac{h}{T^{2}}\theta_{k+1}=a_{k}$$

which has the same form of the tridiagonal system *a_i_x_i_*_−1_ + *b_i_x_i_* + *c_i_x_i_*_+1_ = *d_i_* (see Equation (8)) which is usually solved by applying the Thomas algorithm. It is worth noting that the coefficients of the tridiagonal system described by Equation (A5) are constant only for the linear case.

## References

[1] Landau L.D., Lifshitz E.M. (1976). Mechanics.

[2] Stocker J.J. (1966). Nonlinear Vibrations in Mechanical and Electrical Systems.

[3] Corben H.C., Stehele P. (1960). Classical Mechanics.

[4] Kalmus H.P. (1978). The inverted pendulum. Am. J. Phys..

[5] Michaelis M.M. (1985). Stroboscopic study of the inverted pendulum. Am. J. Phys..

[6] Friedman M.H., Campana J.E., Yergey A.L. (1982). The inverted pendulum: A mechanical analog of the quadrupole mass filter. Am. J. Phys..

[7] Smith H.J.T., Blackburn J.A. (1992). Experimental study of an inverted pendulum. Am. J. Phys..

[8] Lee H., Jung S. (2012). Balancing and navigation control of a mobile inverted pendulum robot using sensor fusion of low cost sensors. Mechatronics.

[9] Sivaraman E., Arulselvi S. (2012). Modelling of an inverted pendulum based on fuzzy clustering technique. Int. J. Comp. Appl..

[10] Park J.H., Kim K.D. Biped Robot Walking Using Gravity-Compensated Inverted Pendulum Mode and Computed Torque Control.

[11] Anderson C.W. (1989). Learning to control an inverted pendulum using neural networks. IEEE Contr. Syst. Mag..

[12] Huang S.J., Huang C.L. (1994). Control of an inverted pendulum using grey prediction model. IEEE Trans. Ind. Appl..

[13] Loram I.D., Lakie M. (2002). Human balancing of an inverted pendulum: Position control by small, ballistic-like, throw and catch movement. J. Physiol..

[14] Macpherson J.M., Horak F.B., Kandel E.R., Schwartz J.H., Jessell T.M. (2000). Posture. Principles of Neural Science.

[15] Guelton K., Delprat S., Guerra T.M. (2008). An alternative to inverse dynamics joint torques estimation in human stance based on Takagi-Sugeno unknown-inputs observer in the descriptor form. Contr. Eng. Pract..

[16] Pinter I.J., van Swigchem R., van Soest A.J., Rozendaal L.A. (2008). The dynamics of postural sway cannot be captured using a one-segment inverted pendulum model: A PCA on segment rotations during unperturbed stance. J. Neurophysiol..

[17] Creath R., Kiemel T., Horak F., Peterka R., Jeka J. (2005). A unified view of quiet and perturbed stance: simultaneous co-existing excitable modes. Neurosci. Lett..

[18] Fuschillo V.L., Bagalà F., Chiari L., Cappello A. (2012). Accelerometry-based prediction of movement dynamics for balance monitoring. Med. Biol. Eng. Comput..

[19] Bagalà F., Fuschillo V.L., Chiari L., Cappello A. (2012). Calibrated 2D angular kinematics by single-axis accelerometers: From inverted pendulum to n-link chain. IEEE Sens. J..

[20] Sabatini A.M. (2011). Estimating three-dimensional orientation of human body part by inertial/magnetic sensing. Sensors.

[21] Bagalà F., Klenk J., Cappello A., Chiari L., Becker C., Lindemann U. (2012). Quantitative description of the Lie-to-Sit-to-Stand-to-Walk transfer by a single body-fixed sensor. IEEE Trans. Neural. Syst. Rehabil. Eng..

[22] Rotenberg D., Slycke P.J., Veltink P.H. (2007). Ambulatory position and orientation tracking fusing magnetic and inertial sensing. IEEE Trans. Biomed. Eng..

[23] Cooper G., Sheret I., McMillian L., Siliverdis K., Sha N., Hodgins D., Kenney L., Howard D. (2009). Inertial sensor-based knee flexion/extension angle estimation. J. Biomech..

[24] Dejnabadi H., Jolle B.M., Casanova E., Fua P., Aminian K. (2006). Estimation and visualization of sagittal kinematics of lower limbs orientation using body-fixed sensors. IEEE Trans. Biomed. Eng..

[25] Kamen G., Patten C., Duke D.C., Sison S. (1998). An accelerometry-based system for the assessment of balance and postural sway. Gerontology.

[26] Moe-Nilssen R. (1998). A new method for evaluating motor control in gait under real-life environmental conditions. Part 1: The instrument. Clin. Biomech..

[27] Mayagoitia R.E., Lötters J.C., Veltink P.H., Hermens H. (2002). Standing balance evaluation using a triaxial accelerometer. Gait Posture.

[28] Mayagoitia R.E., Nene A.V., Veltink P.H. (2002). Accelerometer and rate gyroscope measurement of kinematics: An inexpensive alternative to optical motion analysis systems. J. Biomech..

[29] Lyons G.M., Culhane K.M., Hilton D., Grace P.A., Lyons D. (2005). A description of an accelerometer-based mobility monitoring technique. Med. Eng. Phys..

[30] Williamson R., Andrews B.J. (2001). Detecting absolute human knee angle and angular velocity using accelerometers and rate gyroscopes. Med. Biol. Eng. Comput..

[31] Thomas L.H. (1949). Elliptic Problems in Linear Differential Equations over a Network.

[32] Kalman R. (1960). A new approach to linear filtering and prediction problems. Trans. ASME.

[33] Welch G., Bishop G. (1995). An Introduction to the Kalman Filter.

[34] Salem G.J., Salinas R., Harding F.V. (2003). Bilateral kinematic and kinetic analysis of the squat exercise after anterior cruciate ligament reconstruction. Archive Phys. Med. Rehab..

[35] Arun K., Huang T., Blostein S. (1987). Least-squares fitting of two 3-D point sets. IEEE Trans. Pattern Anal. Mach. Intell..

[36] Hanson R., Norris M. (1981). Analysis of measurements based on the singular value decomposition. SIAM J. Sci. Stat. Comp..

